# SJP-L-5 inhibits HIV-1 polypurine tract primed plus-strand DNA elongation, indicating viral DNA synthesis initiation at multiple sites under drug pressure

**DOI:** 10.1038/s41598-018-20954-5

**Published:** 2018-02-07

**Authors:** Xing-Jie Zhang, Rui-Rui Wang, Huan Chen, Rong-Hua Luo, Liu-Meng Yang, Jing-Ping Liu, Han-Dong Sun, Hong-Bin Zhang, Wei-Lie Xiao, Yong-Tang Zheng

**Affiliations:** 1 Key Laboratory of Bioactive Peptides of Yunnan Province/Key Laboratory of Animal Models and Human Disease Mechanisms of the Chinese Academy of Sciences, Kunming Institute of Zoology, Chinese Academy of Sciences, Kunming, Yunnan 650223 China; 2 Key Laboratory of Medicinal Chemistry for Natural Resource, Ministry of Education and Yunnan Province, Yunnan University, Kunming, Yunnan 650091 China; 3 Kunming College of Life Science, University of Chinese Academy of Sciences, Kunming, Yunnan 650204 China; 4 College of Pharmaceutical Science, Yunnan University of Traditional Chinese Medicine, Kunming, Yunnan 650500 China; 5 State Key Laboratory of Phytochemistry and Plant Resources in West China, Kunming Institute of Botany, Chinese Academy of Sciences, Kunming, Yunnan 650201 China; 6 KIZ-SU Joint Laboratory of Animal Models and Drug Development, College of Pharmaceutical Sciences, Soochow University, Suzhou, Jiangsu 215006 China

## Abstract

In a previous study the small molecule SJP-L-5 that inhibits HIV replication, has been shown to block uncoating of the viral capsid. Continued study showed that SJP-L-5 might hinder HIV capsid uncoating by blocking the completion of reverse transcription. However, to date, the mechanism has not been fully elucidated. Here, the effects of SJP-L-5 for reverse transcription were explored via quantitative PCR, DIG-labelled ELISA, fluorescent resonance energy transfer, and Southern blot assays. We also analyzed the resistance profile of this compound against reverse transcriptase. Our results show that SJP-L-5 preferentially inhibits PPT primed plus-strand DNA synthesis (EC_50_ = 13.4 ± 3.0 μM) over RNA primed minus-strand DNA synthesis (EC_50_ > 3,646 μM), resulting in formation of five segmented plus-strand DNA and loss of HIV DNA flap, suggesting failure of both nuclear import and integration. Moreover, resistance study evidenced that SJP-L-5 requires the amino acid residues Val108 and Tyr181 to exert an inhibitory effect. These results indicate SJP-L-5 as a new non-nucleoside reverse transcriptase inhibitor that inhibits HIV-1 polypurine tract primed plus-strand DNA synthesis, initiating HIV-1 down-stream plus-strand DNA synthesis at multiple sites under drug pressure.

## Introduction

Retroviruses (i.e., human immunodeficiency virus, HIV) are single-stranded RNA viruses that infect eukaryotic cells. The retroviral life cycle is characterized by reverse transcription (RT) of the single-stranded plus RNA genome and integration of the complementary DNA (cDNA) into the host genome. RT is a key step in HIV replication, and this process is responsible for the synthesis of a double-strand DNA from the viral single-strand RNA genome^1^.

**Figure 1:**
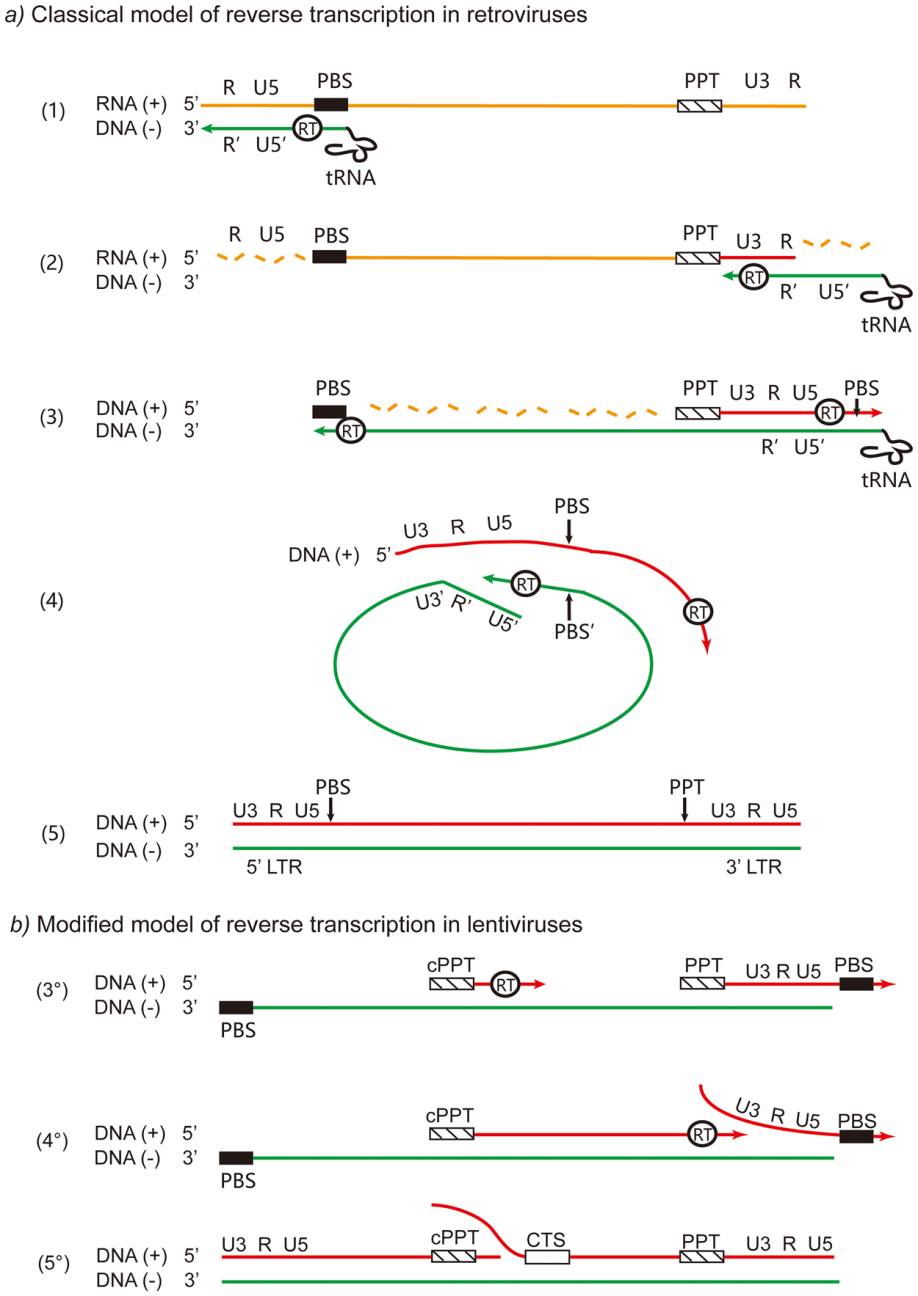
Different processes of reverse transcription in retroviruses. (**a**) Classical model of reverse transcription in retroviruses. (1) Reverse transcription is initiated by a tRNA primer at the PBS site near the 5′ end of the genome. (2) R′U5′ is translocated to the 3′ end of the genome and triggers the minus-DNA synthesis. This step is known as the first jump. (3) PPT, near the 3′ end of the genome, is used as a primer to initiate the plus-strand DNA synthesis. (4) PBS is used as a primer to form a circular DNA structure; this step is known as the second jump. (**b**) Modified model of reverse transcription in lentiviruses (i.e., HIV). HIV has an extra PPT site in the center of the genome, called cPPT. (3°) Both cPPT and PPT are used as primers to initiate the plus-strand DNA synthesis. (4°) The downstream plus-strand DNA is synthesized until the RTase reaches a strong-stop DNA site (U3-R-U5). (5°) Finally, the synthesis of the upstream plus-strand DNA stops at the CTS site near the center of the genome, and a discontinued plus-strand DNA is formed. Note that the actual proportions of the sequences have been altered in the diagram. Yellow line: viral plus-strand RNA; green line: viral minus-strand DNA; red line: viral plus-strand DNA. This figure was modified with permission from REF. 2© (2017) Microbiology Society.

RT is a complex process in which reverse transcriptase (RTase) has three functions and makes two jumps^2^. These three RTase functions include: (1) RNA-dependent DNA polymerization (RDDP) activity, converting single-stranded viral RNA to minus DNA; (2) DNA-dependent DNA polymerization (DDDP) activity, converting minus DNA to plus DNA; (3) RNase H activity, digesting RNA from RNA/DNA hybrids^3^. The first RTase jump is triggered by a minus-strand strong-stop DNA (−sssDNA), which is used as a primer to synthesize a large minus-DNA fragment. The second jump is triggered by the plus-strand strong-stop DNA (+sssDNA) near the 3′ end of the RNA genome, synthesized from the 3′ polypurine tract (PPT), which is used as a primer. After these two jumps, three types of viral DNA have been synthesized: linear DNA, long-terminal repeat (LTR) DNA, and 2-LTR DNA (Fig. 1). Unlike other retroviruses (i.e., MMV or AMV), HIV, as a lentivirus, has a PPT sequence in the center of the RNA genome (central PPT or cPPT), as well as in the integrase gene^4^. Previous studies suggested that the cPPT forms a gap called flap in the center of the linear DNA during RT. Thus, plus DNA of the HIV genome is discrete and holds a triple DNA structure in the center that is essential for importing the pre-integrated complex into the nucleus^5^. Hence, this DNA flap is a potential target of anti-HIV drugs; however, such inhibitors are rarely reported. A DNA flap inhibitor could also help understanding the late process of reverse transcription, as well as the early steps of nuclear import.

Since the first RTase inhibitor, zidovudine (AZT), was approved by the FDA three decades ago, RTase has become a major target in highly active antiretroviral therapy (HAART) against HIV infection^6^. Unlike nucleoside RTase inhibitors (NRTIs), non-nucleoside RTase inhibitors (NNRTIs) bind to the hydrophobic bag and inhibit its polymerase activity by an allosteric effect. Normally, NNRTIs inhibit both RNA- and DNA-dependent DNA polymerization activities, but not the ribonuclease H (RNase H) activity.

**Figure 2:**
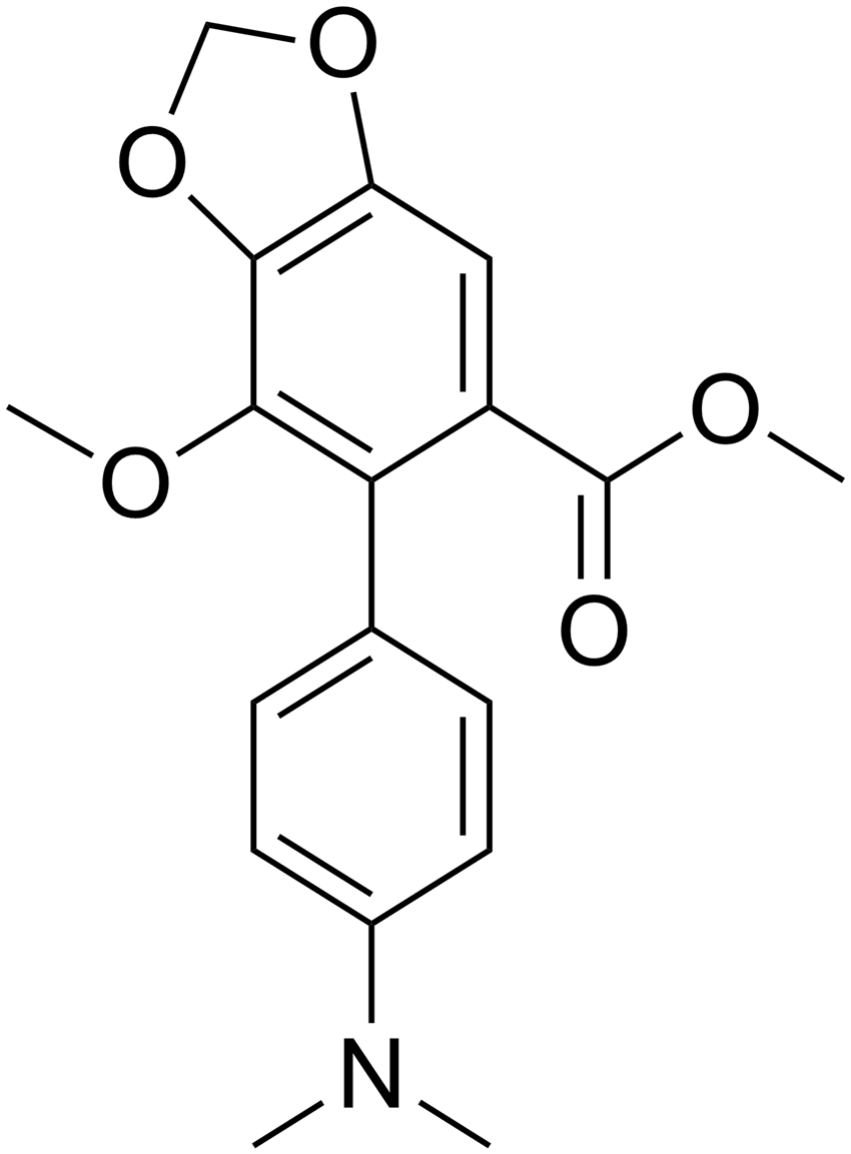
Structure of SJP-L-5.

Our previous study showed that SJP-L-5 (Fig. 2), a nitrogen-containing biphenyl compound, whose synthesis was based on dibenzocyclooctadienelignan, gomisin M2 (SM-10), blocks the nuclear entry of the HIV pre-integrated complex by inhibiting capsid uncoating^7^. However, the mechanism with which SJP-L-5 blocks the uncoating of the viral capsid remains unknown. Our data (unpublished) suggested that SJP-L-5 may inhibit the RTase DNA-dependent DNA polymerase function. Therefore, we hypothesize that SJP-L-5 inhibits the viral plus-strand DNA synthesis by hindering full-length plus-strand DNA maturation.

Taking our hypothesis into account, we examined the molecular mechanisms of SJP-L-5 inhibition. Here, we present new data on SJP-L-5 and its inhibitory activity on the three RTase functions. We also analyze the effect of SJP-L-5 on the synthesis of viral plus- and minus-strand DNA. Finally, we show a viral resistance profile of SJP-L-5 against HIV-1_IIIB_.

## Results

### SJP-L-5 does not block the initiation of reverse transcription, but partially decreases late-RT products in HIV-1_IIIB_-infected C8166 cells

In our previous study, we showed that the antiviral target of SJP-L-5 is the uncoating of the capsid, based on single-round pseudotyped virus investigation^7^. Completion of RT is believed to trigger capsid disassembling^8^. To explore the stage at which this compound inhibits multi-round replicative viruses, real-time qPCR assays were carried out. The -sssDNA is a signal of RT initiation, which involves RTase RDDP activity. On the other hand, late-RT DNA is a signal of late RT, which involves the RTase DDDP activity. Dead-end circular 2-LTR DNA is a nuclear import signal.

**Figure 3:**
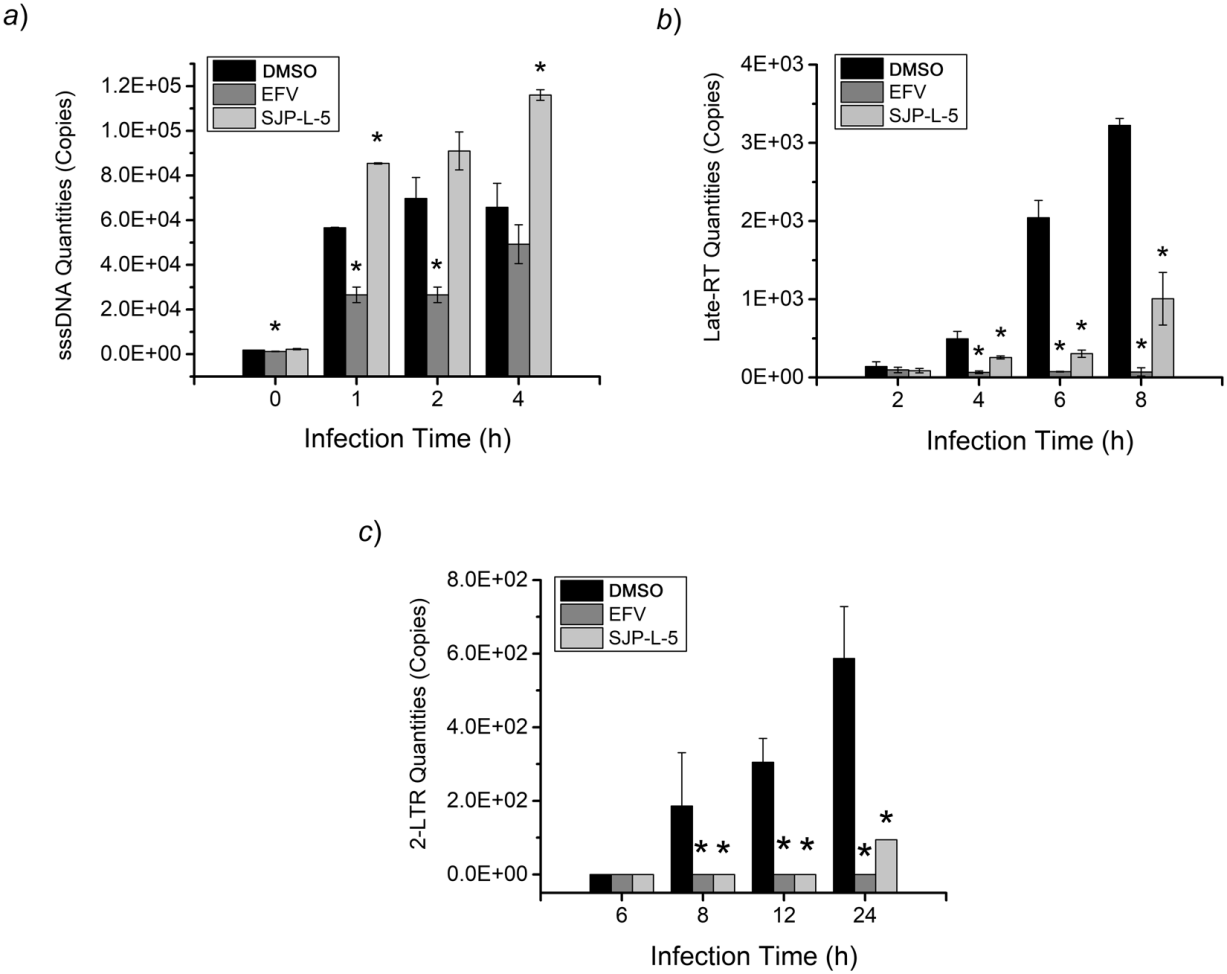
Real-time qPCR assays of HIV-1 viral DNA levels in SJP-L-5-treated cells. HIV-1_IIIB_-infected C8166 cells were co-cultured with SJP-L-5 (100 μM), EFV (200 nM) or DMSO (0.5%, *v/v*) during different time points up to 24 h. Viral DNA was isolated and quantified by real-time qPCR. (**a**) sssDNA formation was not inhibited with SJP-L-5, and these molecules significantly accumulated at 1 and 4 h after HIV-1 infection (*P* < 0.05). (**b**) Late-RT quantities were significantly reduced by SJP-L-5 at 4, 6, and 8 h (*P* < 0.05). (**c**) 2-LTR quantities were significantly reduced with SJP-L-5 at 8, 12, and 24 h (*P* < 0.05).

In the first 4 h, -sssDNA levels were not reduced by SJP-L-5, at a concentration sufficient to block the HIV-1 infection in cell-based assays (100 μM) (Fig. 3a). However, late-RT DNA products were partially reduced by this compound from 4 to 8 h (Fig. 3b), and 2-LTR levels were totally inhibited by SJP-L-5 from 8 to 12 h (Fig. 3c). The positive control (EFV) reduced the levels of the three viral DNA forms, suggesting that it inhibits the early steps of RT. Taken together, these results show that SJP-L-5 blocks HIV-1 infection during the late process of RT.

### SJP-L-5 inhibits DDDP activity in a dose-dependent response

Based on the data shown in Fig. 3, we predicted that SJP-L-5 would target the DDDP. To evaluate this assumption, we measured the three functions of the RTase. We conducted a DIG-labelled dUTP ELISA to evaluate the RDDP activity, and a fluorescent-labelled dUTP assay to detect the DDDP or the RNase H activities.

**Figure 4:**
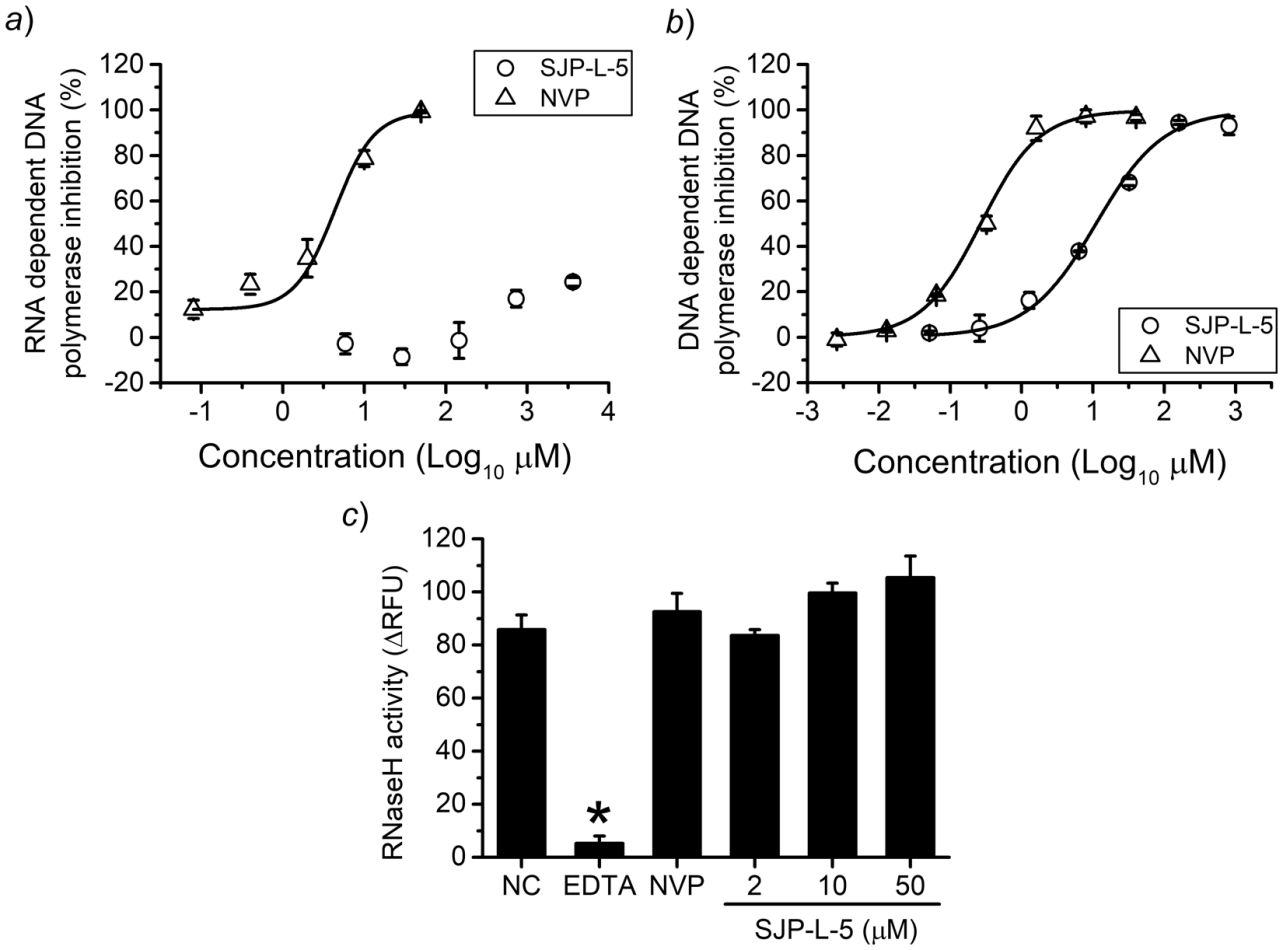
Reverse transcriptase activity assay. (**a**) The RNA-dependent DNA polymerase activity was measured using a DIG-labelled oligo(dT) ELISA. The inhibitory rate of SJP-L-5 was less than 25% at 3,646 μM. The EC_50_ of NVP was 3.98 ± 2.26 μM. (**b**) The DNA-dependent DNA polymerase activity was measured using an AF-488-labelled DNA FRET assay. The EC_50_ of SJP-L-5 and NVP were 13.4 ± 3.0 μM and 342 ± 35 μM, respectively. (**c**) The RNase H activity was measured using a fluorescein-labelled RNA FRET assay. Neither NVP (200 nM) nor SJP-L-5 (2, 10, and 50 μM) inhibited the RNase H activity, but EDTA (20 mM) significantly reduced it (*P < *0.05).

As shown in Fig. 4a, the RTase RDDP activity was barely inhibited at the highest concentration of SJP-L-5 (inhibitory rate <25% at 3,646 μM) but was efficiently blocked by NVP with an EC_50_ of 3.98 ± 2.26 μM in a dose-dependent manner, suggesting that the RDDP activity is not the main target of SJP-L-5. However, the DDDP activity was inhibited by both SJP-L-5 and NVP in a dose-dependent manner, displaying an EC_50_ of 13.4 ± 3.0 μM and 342 ± 35 nM, respectively (Fig. 4b). This result shows that SJP-L-5 is an inhibitor of the RTase DDDP activity and, thus, may interfere with viral plus-DNA synthesis. Neither SJP-L-5 nor NVP inhibited the RNase H activity (Fig. 4c).

### SJP-L-5 accumulates different-length viral downstream plus-strand DNA delaying viral DNA maturation

Next, we assessed the impact of SJP-L-5 on viral plus-strand DNA synthesis and DDDP activity by treating the cells with this inhibitor. DNA flap is a viral plus-strand DNA that is discrete when the RT just finished^9,10^. Based on this feature, Southern blot analysis in denaturing conditions was carried out. Two different probes were used, namely, a double-stranded DNA probe within the RTase gene next to the genomic 5′ end and a downstream plus-strand DNA probe within the gp41 gene next to the genomic 3′ end.

**Figure 5:**
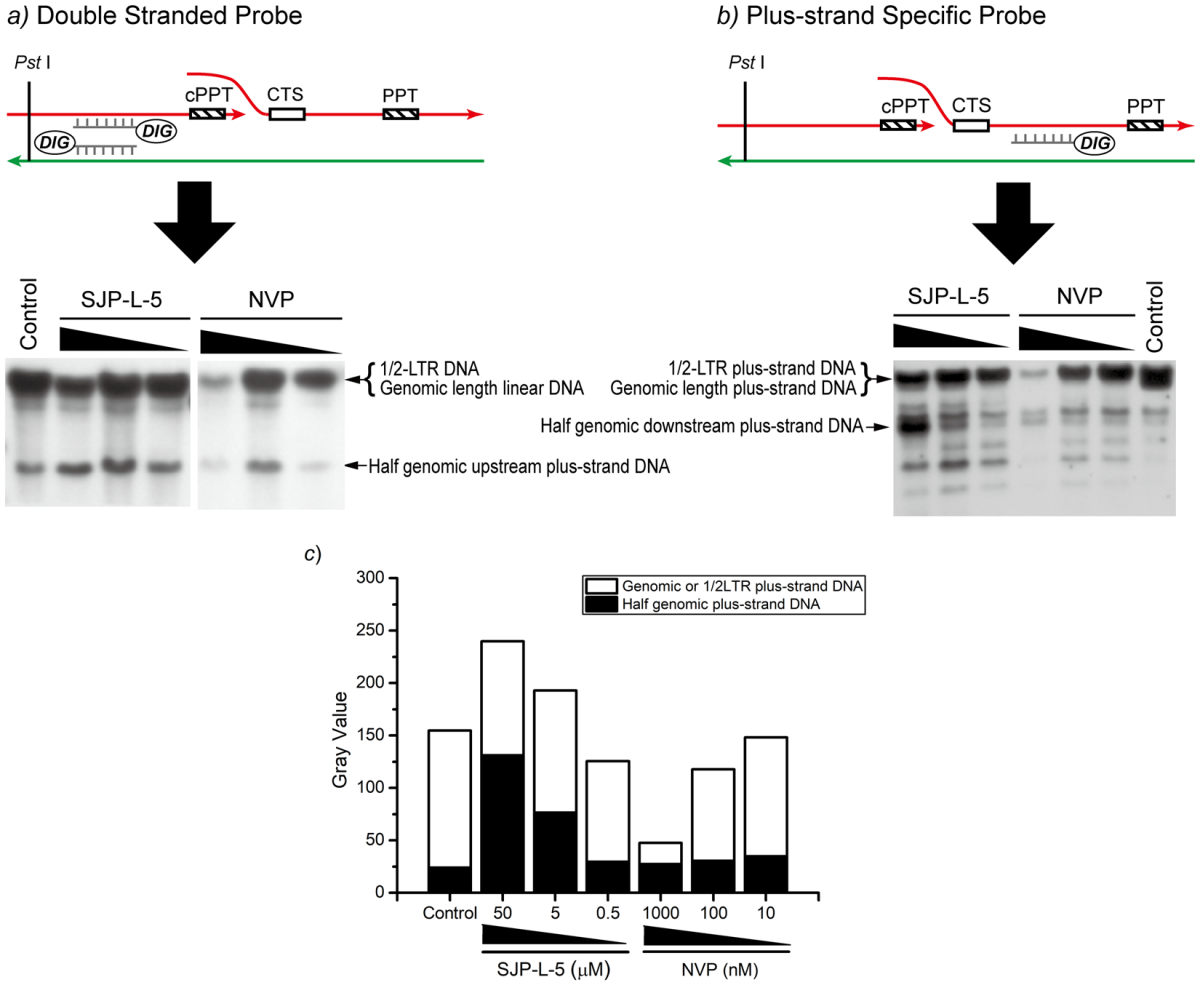
Structural analysis of viral DNA via Southern blot. HIV-1_IIIB_-infected C8166 cells were co-cultured with SJP-L-5 (50, 5, and 0.5 μM) or NVP (1000, 100, and 10 nM) for 48 h. Viral DNA was extracted using Hirt′s method and denatured by boiling in a water bath. The DNA samples were digested with *Pst* I, subjected to electrophoresis in 0.7% agarose gel with 0.5×TBE and hybridized using DIG-labelled probes. (**a**) Southern blot with double-stranded DNA detection. The upstream half-genome levels of the plus-strand DNA were not reduced with SJP-L-5 but were reduced with NVP. (**b**) Southern blot with downstream plus-strand DNA-specific detection. The downstream half-genome levels of the plus-strand DNA were accumulated with SJP-L-5, possibly due to a delay in full-length viral DNA maturation, displaying a different mechanism from that observed with NVP. RAL (1 µM) was used to prevent integration of viral DNA into host chromosomes in all groups. Full-length blots are presented in Supplementary Figure S1. (**c**) Gray analysis of down-stream plus-strand DNA probe hybridization. Half genomic plus-strand DNA of SJP-L-5 accumulated in a dose-responsive manner, but was maintained at a base level of NVP at different concentrations. Furthermore, the total amounts of viral DNA were accumulated via SJP-L-5 in treatment with 50 and 5 μM. The genomic or 1/2LTR plus-strand DNAs were not affected by SJP-L-5 in a series of concentrations. The total amounts of viral DNA and genomic or 1/2LTR plus-strand DNAs decreased by NVP in a dose-responsive manner.

The upstream half-genome of the plus- and minus-strand DNA was detected with a double-stranded DNA probe in SJP-L-5- or NVP-treated cells. Both plus- and minus-strand DNA levels remained unaltered at increasing concentrations of SJP-L-5 (Fig. 5c). Moreover, upstream plus-strand DNA was higher than the control, suggesting that SJP-L-5 would not block upstream plus-strand DNA synthesis. On the other hand, plus- and minus-strand DNA levels decreased with NVP in a dose-dependent manner, suggesting that NVP inhibits both types of DNA (plus and minus). Downstream plus-strand DNA levels, detected with a plus-strand DNA probe, accumulated with SJP-L-5 in a dose-dependent manner **(**Fig. 5c), exhibiting at least five different DNA lengths (Fig. 5b), suggesting that SJP-L-5 induces multiple initiation sites to start the downstream plus-strand DNA synthesis while usually only the cPPT site is used as a primer. These results demonstrate that SJP-L-5 delays viral downstream plus-strand DNA maturation, disturbing formation of a triple-DNA flap.

### SJP-L-5 induces mutation of the RTase gene in genotypic resistance assays

To further investigate the characteristics of SJP-L-5 inhibition of the RTase, we selected resistant viruses from HIV-1_IIIB_-infected C8166 cells exposed to increasing concentrations of SJP-L-5, and sequenced the RTase gene for a genotypic assay. Since it is very difficult to introduce a mutation in a live virus, we used the plasmid pNL4-3 (the RTase DNA sequence of IIIB and NL4-3 are highly similar with a similarity >99%) as a tool to perform site-directed mutations.

**Figure 6:**
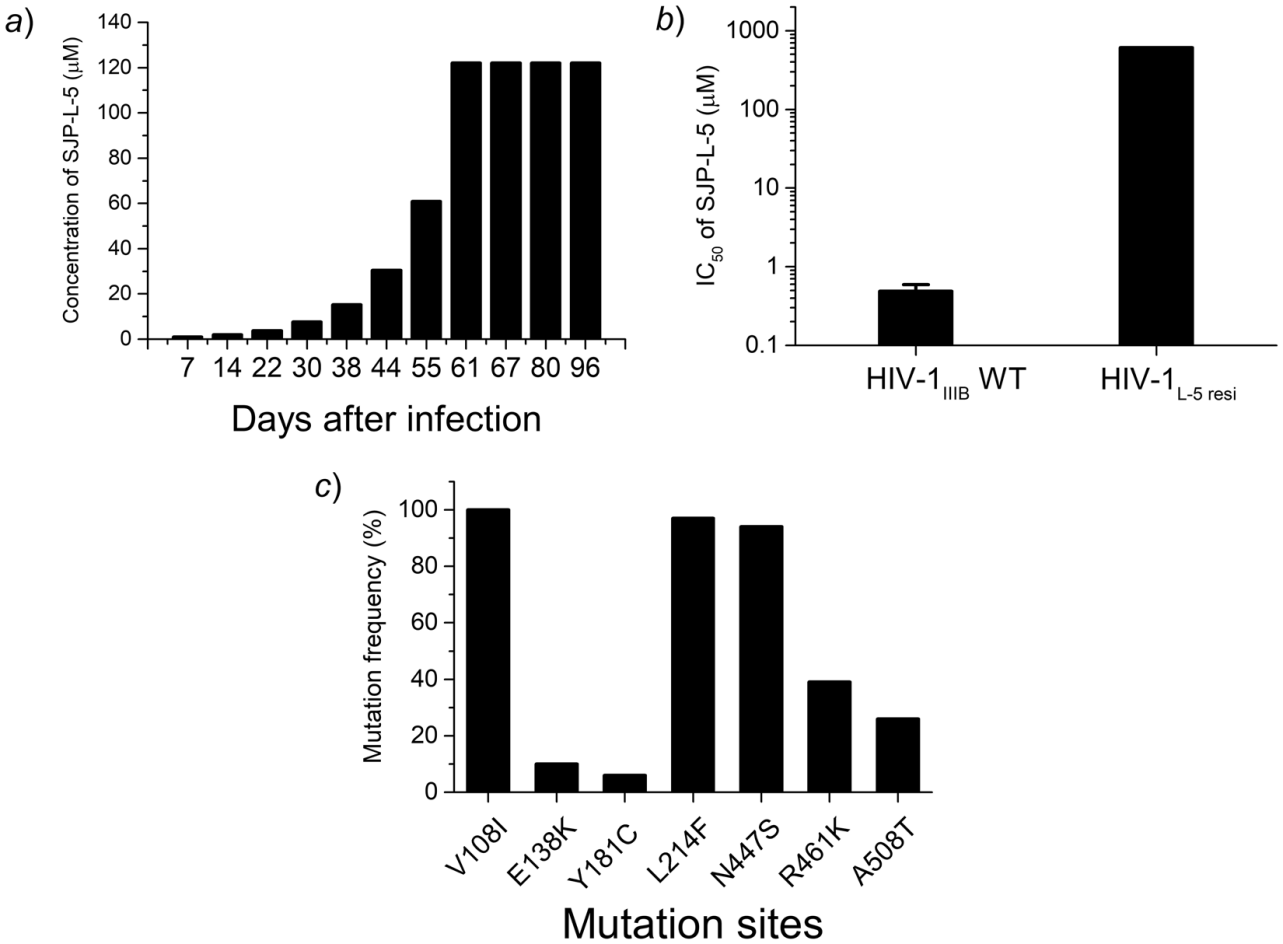
Induction of an SJP-L-5-resistant virus strain. A resistant virus strain to SJP-L-5 was selected by adding increasing concentrations of SJP-L-5 (0.9 to 122 μM) to HIV-1_IIIB_-infected C8166 cells. (**a**) SJP-L-5 concentrations during the experiment. After 96 days of induction, a SJP-L-5-resistant virus strain was selected. (**b**) Antiviral activity of SJP-L-5 against the induced resistant virus. The selected resistant virus showed an IC_50_ 1240-fold higher than the wild-type value. (**c**) Genotypic patterns of HIV-1_IIIB_ selected with SJP-L-5. The viral RNA was reverse transcribed, cloned into a TA cloning vector, and sequenced. Seven mutation sites were discovered; three of which are hot spots (V108I, L214F, and N447S).

SJP-L-5 concentrations were increased from 0.9 to 122 μM during 11 passages to select resistant HIV-1_IIIB_ viruses (Fig. 6a). After a 96-day selection, an SJP-L-5 resistant strain was obtained: HIV-1_L-5resi_. The EC_50_ of SJP-L-5 against HIV-1_L-5resi_ was above 608 μM (1240-fold higher than the wild-type, Fig. 6b) and higher than the 122 μM value obtained in the virus selection assay, suggesting we successfully obtained a SJP-L-5-resistant virus strain. Afterwards, the DNA of HIV-1_L-5resi_-infected C8166 cells was extracted and the full-length RTase sequence (1680 bp) was analyzed. The sequence analysis revealed that seven amino acids were mutated within the RTase gene, namely, V108I, E138K, Y181C, and L214F within the DNA polymerase motif, and N447S, R461K, and A508T within the RNase H motif (Fig. 6c). These results show that SJP-L-5 induces gene mutations and supports the idea which the RTase is a target of this inhibitor.

### SJP-L-5 exhibits diminished activity against NNRTIs resistant mutants, but is sensitive to NRTIs resistant mutants in phenotypic resistance assays

To understand the interactions between the RTase and SJP-L-5, we investigated whether SJP-L-5 would be less sensitive to RTase mutants. Three single-site mutants (V108I, E138K, and Y181C, located in the polymerase domain), which were indicated from genotypic resistance assays, were used for a phenotypic resistance assay with SJP-L-5. Additionally, other known resistant viruses (HIV-1_A17_, HIV-1_74V_, and HIV-1_A018_) were tested. As shown in Table 1, SJP-L-5 was resistant to NNRTI mutations, including V108I, E138K, Y181C, and “K103N + Y181C”, with fold changes (FC) ranging from 25 to 158. However, SJP-L-5 was sensitive to NRTI mutations (HIV-1_L74V_ and HIV-1_AO18_, with FC of 0.8 and 1.4, respectively). NVP displayed similar antiviral characteristics to SJP-L-5 against mutants and resistant viruses. In summary, our results suggest that SJP-L-5 is an NNRTI. However, it exerts a novel mechanism as SJP-L-5 preferentially inhibits the RTase DDDP activity instead of the RDDP.

**Table 1:** Anti-HIV-1 activity of SJP-L-5 against mutated viruses^a^.

RTase-mutated viruses	Compounds			
	SJP-L-5		NVP	
	EC_50_ (μM)^b^	FC^c^	EC_50_ (nM)	FC
WT (HIV-1_NL4-3_)	0.53 ± 0.12	NA^d^	11.97 ± 0.69	NA
V108I	13.01 ± 2.28	25	433.26 ± 63.14	36
Y181C	23.70 ± 13.01	45	4,896 ± 2,293	409
E138K	17.84 ± 0.38	34	175.65 ± 20.72	15
HIV-1_A17_ (K103N + Y181C)	78.2 ± 13.5	148	203.4 ± 8.1 (μM)	16,992
HIV-1_74V_ (L74V)	0.44 ± 0.05	0.8	35.5 ± 2.4	3.0
HIV-1_AO18_ (TAMS^e^)	0.74 ± 0.07	1.4	32.4 ± 9.2	2.7

^a^Abbreviations: WT, wild-type; NVP, nevirapine.

^b^EC_50_s are exhibited by means ± standard deviations, n ≥ 3.

^c^FC, fold change (calculated as the mean EC_50_ of mutants divided by the wild-type HIV-1_NL4-3_ value).

^d^NA, not applicable.

^e^TAMS, thymidine analog mutations, containing azidothymidine-resistant sites.

## Discussion

To date, there are five NNRTIs in clinical use, including nevirapine, delavirdine, efavirenz, etravirine, and rilpivirine. These drugs inhibit viral minus-strand DNA synthesis^11–15^. Here, we describe a novel compound, SJP-L-5, which inhibits the plus-strand DNA elongation rather than minus-strand DNA synthesis, presenting a different mechanism from that utilized by marketed drugs.

The synthesis of the minus-strand DNA is initiated by a tRNA primer, which binds to the primer-binding site (PBS) near the 5′ end of the viral genomic RNA. Initiation of RT is conducted by RTase RDDP activity from the tRNA primer, and the synthesis of -sssDNA is one of the first products of this process^16^. Real-time qPCR assays showed that SJP-L-5 does not reduce the -sssDNA products in the first 4 h, but reduces late-RT and 2-LTR DNA levels (Fig. 3a–c). These results demonstrate that SJP-L-5 blocks HIV-1 infection through RT inhibition, although it does not work early in this process. This mechanism is quite different from those previously reported since all FDA-approved NNRTIs target the RDDP activity while SJP-L-5 inhibits the DDDP activity^6,17^.

Recently, the emergence of drug resistance has become a major concern in antiviral therapy^1,18,19^. To overcome this issue, on one hand, a series of novel structural compounds were designed, such as rilpivirine and dolutegravir^15,20^; on the other hand, new drug targets are gaining more attention, for example, RNase H of RTase^21,22^. To understand the mechanisms with which SJP-L-5 blocks RTase, we analyzed the three functions of the viral enzyme with methods that include ELISA and FRET. SJP-L-5 inhibited the RTase DDDP activity in a dose-dependent manner with an EC_50_ of 13.4 ± 3.0 μM. Strikingly, this compound showed little inhibition of the RDDP activity, which is distinctive from other NNRTIs. RNase H inhibition was not observed either (Fig. 4c). Cell-based qPCR assays suggested that SJP-L-5 preferentially inhibits plus-strand DNA synthesis rather than minus-strand DNA formation. These interesting results prompted the question of the mechanism of this inhibition. We next focused on the HIV DNA flap, a special DNA structure that exists in lentiviruses and is formed by discontinuous plus-strand DNA synthesis.

Two decades ago, it was reported that HIV reverse-transcribed DNA is discontinuous and bears two initiation sites to synthesize the viral plus-strand DNA^4,9,10^. Later, researchers found that a triple-DNA structure formed by the discontinuous plus-strand, called DNA flap, contributes to the nuclear import of the HIV-1 genome^5^. DNA flap was then believed as a viral promoting element for the uncoating of HIV-1 at the nuclear pore^23^. In recent years, RT and uncoating seem to be sequential processes since RT influences uncoating kinetics. DNA-RNA hybrid molecules may form a more rigid extended structure, which in turn could provide the outward force to destabilize the capsid during the early steps of RT^8^. Our published data demonstrated that SJP-L-5 blocks viral DNA nuclear entry by interrupting capsid disassembling^7^. In this paper, we analyzed the late process of RT by Southern blot. DNA hybridization data showed that SJP-L-5 does not inhibit the minus-strand DNA formation but impairs downstream plus-strand DNA elongation (Fig. 5). These results suggest that SJP-L-5 is a special RTase inhibitor that blocks the central DNA flap to be linearized by retaining the viral downstream plus-strand DNA at half genomic length. In answer to our previous question, we described the viral capsid uncoating delay in the SJP-L-5 treatment group. Due to the novel mechanism underlying SJP-L-5 inhibition, it was not possible to discover its role in our previous study^7^. From our experience, we strongly suggest that the three functions of the RTase are evaluated during antiviral drug screening.

NNRTIs are small hydrophobic molecules that inhibit the HIV-1 RTase by an allosteric effect. They bind to a pocket located about 10 Å away from the dNTP binding site^24^. We used resistance assays, including resistance selection and mutant sensitivity assays, to explore the interactions between SJP-L-5 and HIV-1 RTase. Since this compound did not inhibit RNaseH, we did not further evaluate the RNaseH mutations (N447S, R461K, and A508T) with SJP-L-5. Moreover, L214F occurs naturally as a polymorphism and does not cause NNRTI resistance^25^. The results showed that SJP-L-5 is resistant to V108I, E138K, Y181C, and “K103N + Y181C”, which are traditional resistance sites of NNRTIs^26^; the main sites being V108I and Y181C, based on genotypic and phenotypic assays. These results suggest that SJP-L-5 may bind to Val108 and Tyr181 more firmly than to other amino acids. Therefore, SJP-L-5 binds to the HIV-1 RTase hydrophobic bag in the same way as NNRTIs. However, our study shows that SJP-L-5 preferentially inhibits RTase DDDP activity. RT needs to be further investigated and, to this regard, SJP-L-5 could be a valuable tool to understand this process.

Multiple segmented plus-strand DNA is rarely reported in HIV but was observed in another retrovirus: the avian sarcoma-leukosis virus (ASLV), which is an *alpharetrovirus* genus virus^27^. Previous studies showed that only one gap (a triple DNA flap) exists in the center of HIV DNA genome^4,5,9^. Our results suggest that more than one gap may exist under the treatment of SJP-L-5. This phenomenon provides insight into the details of HIV reverse transcription. However, why segmented plus-strand DNA only formed under the pressure of SJP-L-5 needs to be further investigated.

**Figure 7:**
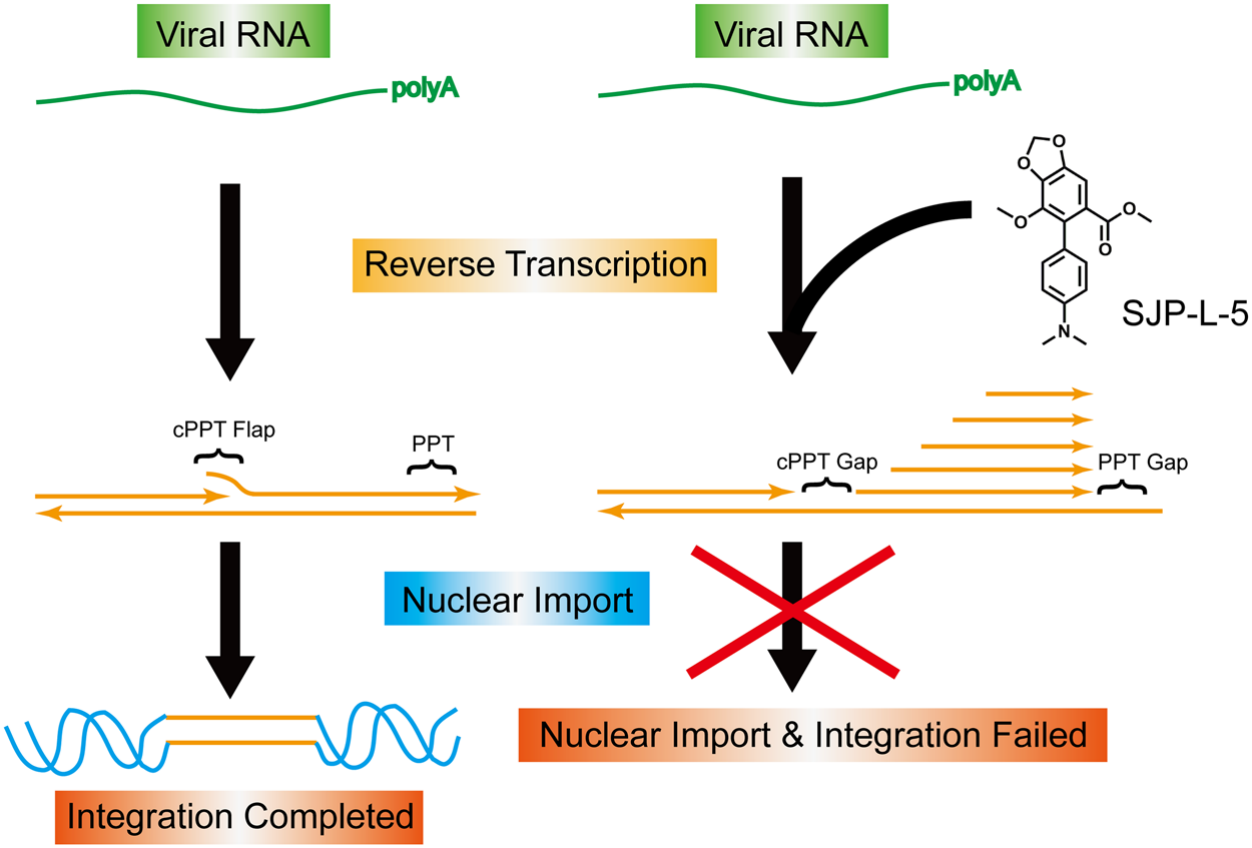
A Model of how SJP-L-5 inhibits PPT-primed HIV-1 plus-DNA synthesis. HIV-1 plus-strand DNA synthesis is initiated by cPPT or PPT in the central genome or 3′ end, leading to formation of a triple-strand flap in the center. Later, the viral DNA genome enters the nucleus and is integrated into the host DNA genome. In response to SJP-L-5 treatment, both cPPT and PPT primed plus-strand DNA synthesis are blocked, leading to a cPPT gap and a PPT gap in the center and the 3′ end of HIV-1 DNA genome. Under the pressure of this compound, HIV-1 uses multiple sites to initiate its plus-strand DNA synthesis within down-stream plus-strand DNA, forming five segmented plus-strand DNA bands and two PPT gaps (cPPT and PPT). Due to a lack of matured DNA (a triple-DNA flap), the impeded viral DNA fails to enter the host cell nucleus and cannot integrate into the host DNA genome.

Based on our results, we suggest a model of how SJP-L-5 inhibits HIV-1 replication (Fig. 7). Typically, the HIV-1 DNA genome is formed during reverse transcription with a triple-DNA flap at the center. This flap has been suggested as a cis-determinant of nuclear import^5^. Under SJP-L-5 treatment, both cPPT and PPT primed DNA dependent DNA synthesis are inhibited. Furthermore, HIV-1 uses multiple sites to initiate DNA replication in the downstream plus-DNA, leading to five lengths of DNA, one cPPT gap, and formation of one PPT gap. Without flap formation, the defective viral genome cannot enter the host cell nucleus, and virus replication is blocked.

In conclusion, SJP-L-5 inhibits the late stage of the RT process and preferentially inhibits plus-strand DNA synthesis over minus-strand DNA, resulting in formation of five segmented plus-strand DNA fragments and deficient HIV DNA flap, suggesting that both nuclear import and integration failed. Moreover, the resistance study evidenced that SJP-L-5 requires the amino acid residues Val108 and Tyr181 to exert its inhibitory effect. These results indicate SJP-L-5 as a new NNRTI that inhibits HIV-1 polypurine tract (PPT) primed plus-strand DNA synthesis, while initiating HIV-1 down-stream plus-strand DNA synthesis at multiple sites under drug pressure.

## Materials and Methods

### Compounds and reagents

SJP-L-5, with a molecular weight of 329.13, was provided by Professor Handong Sun (Kunming Institute of Botany, Chinese Academy of Sciences). Nevirapine (NVP) and efavirenz (EFV) were purchased from US Pharmacopeia (USA). All compounds were dissolved in dimethyl sulfoxide (DMSO) and stored at −20 °C. DMSO, Tris, KCl, MgCl_2_, 3-[(3-cholamidopropyl) dimethylammonio]−1-propane sulfonate (CHAPS), dithiothreitol (DTT), and ethylene glycol tetra-acetic acid (EGTA) were purchased from Sigma-Aldrich (USA). dUTP-AF555 was purchased from Invitrogen (USA), and dATP, dCTP, and dGTP were purchased from Takara (China).

### Cells and viruses

C8166 cells were kindly provided by the AIDS Reagent Project, the UK Medical Research Council (MRC). C8166 are human T-lymphoblastoid cells sensitive to HIV infection, exhibiting a rapid and pronounced cytopathic effect. C8166 cells were cultured in RPMI 1640 medium supplemented with 10% (*v*/*v*) fetal bovine serum (FBS) with 100 U/mL penicillin G and 100 μg/mL streptomycin in a humidified incubator with 5% CO_2_ at 37 °C. Laboratory-adapted strains (HIV-1_NL4-3_) and RT-inhibitors resistant strains (HIV-1_A17_, HIV-1_74V,_ and HIV-1_A018_) were obtained from the NIH AIDS Research and Reference Reagent Program (Bethesda, MD, USA). All viruses were propagated in C8166 cells.

### Quantitative PCR of viral reverse transcripts

C8166 cells were seeded at a density of 1 × 10^6^ cells/well in 24-well culture plates and infected with HIV-1_IIIB_ at M.O.I. 0.5 for 2 h under gentle rocking every 15 min at 4 °C. After 2 h of virus adsorption, the cells were washed and synchronized. Then, the cells were incubated in presence or absence of 100 μM SJP-L-5, 200 nM EFV, or 0.5% (*v*/*v*) DMSO. Cells were collected at 0, 1, 2, 4, 6, 8, 12, and 24 h of incubation with the drugs. Total cellular DNA was isolated using Blood Gen Mini Kit (CWBIO, China). The -sssDNA was amplified using hRU5-F2 (5′-GCC TCA ATA AAG CTT GCC TTG A-3′) and hRU5-R (5′-TGA CTA AAA GGG TCT GAG GGA TCT-3′) primers with the hRU5-P probe (5′-FAM-AGA GTC ACA CAA CAG ACG GGC ACA CAC TA-TAMRA-3′). Late reverse transcription product U5ψ (late-RT) was amplified with MH531 (5′-GCC TCA ATA AAG CTT GCC TTGA-3′) and MH532 (5′-GCC TCA ATA AAG CTT GCC TTG A-3′) primers and LRT-P probe (5′-FAM-CAG TGG CGC CCG AAC AGG GA-TAMRA-3′). For 2-LTR circle, the LTR-R3 primer (5′-AGG TAG CCT TGT GTG TGG TAG ATC C-3′), the U5-specific primer (5′-AGG TAG CCT TGT GTG TGG TAG ATC C-3′), and the p-HUS-SS1 probe (5′-FAM-TAG TGT GTG CCC GTC TGT TGT GTG AC-TAMRA-3′) were used. The 20-μL reaction mixture contained 2 μL of sample or standard DNA, and the following final concentrations of 1× Premix Ex Taq (probe qPCR) (Takara), 0.2 μM sense primer, 0.2 μM antisense primer, 0.1 μM probe, and 1× ROX Reference Dye II. PCR was performed using an ABI PRISM 7500 Fast Real-Time PCR System (Applied Biosystems). Cycling conditions included a hot start (95 °C for 2 min), 40 cycles of denaturation (95 °C for 15 s), and an extension steps (60 °C for 30 s).

### RDDP activity

HIV-1 RTase RDDP activity was measured by a DIG-labelled ELISA assay using the Reverse Transcriptase Kit (Roche, Germany), according to manufacturer′s instructions.

### HIV-1_IIIB_ recombinant RT (rRTase) expression and purification

HIV-1_IIIB_ RTase p66 and p51 subunits were cloned into two pET-30a vectors, and recombinant p66- and p51-His subunits were over-expressed in *E. coli* BL21(DE3). The p66/p51-His heterodimer was purified by Ni^2+^ affinity agarose purification as previously described^28^. The rRTase was used for the following two assays.

### PPT-primed DDDP activity

HIV-1 RTase DDDP activity was measured via fluorescent resonance energy transfer (FRET) assay as previously described, with minor modifications^17^. The DNA template (5′-AF488-AGT CCC CCC TTT TCT TTT AAA AAG TGG CTA AGA), and the DNA primer (PPT-based) (5′-TTA AAA GAA AAG GGG GG) were synthesized by Sangon Biotech (China). Briefly, DNA template and DNA primer were annealed at temperatures ranging from 90 °C to room temperature to form 20 μM DNA/DNA hybrids. A dXTP mixture of dATP, dCTP, dGTP, and dUTP-AF555 was prepared at 0.4 μM. rRTase was dissolved in reaction buffer (50 mM Tris, 80 mM KCl, 6 mM MgCl_2_, 0.025% (*m*/*v*) CHAPS, 1 mM DTT, and 0.1 mM EGTA pH 7.8) at 320 ng/mL. The 50-μL enzymatic reaction, preceded by incubation for 15 min at room temperature, contained 320 ng/ml rRTase, 80 nM DNA/DNA hybrids, and the test compounds. The reaction was initiated by adding 50 μL of dXTP to a final volume of 100 μL in a black 96-well plate. The plate was then incubated at room temperature in the dark for 15 min. Finally, the plate was measured using a Flex Station 3 reader (Molecular Device, USA) with excitation at 485 nm and emission at 530 nm (cutoff = 515 nm), and the EC_50_ was calculated.

### RNase H activity

HIV-1 RNase H activity was measured by FRET assay as previously described, with minor modifications^29^. The DNA (DD18) (5′-Dabcyl-AGC TCC CAG GCT CAG ATC) and RNA (FR18) (5′-GAU CUG AGC CUG GGA GCU-fluorescein) fragments were synthesized by Takara (China). Briefly, DD18 and FR18 were annealed at temperatures ranging from 90 °C to room temperature to form 10 μM RNA/DNA hybrids. rRTase was dissolved in reaction buffer (the same as the one used to measure the DDDP activity) at 400 ng/mL. Fifty microliters of a 0.4 μM RNA/DNA hybrid solution was mixed with the compound dilutions to a final concentration of 100 μL in a black 96-well plate. The reaction was initiated by adding 50 μL of rRTase solution and incubated at 37 °C for 1 h. The plate was measured using a Flex Station 3 reader, with excitation at 490 nm and emission at 525 nm (cutoff = 515 nm), and the EC_50_ was calculated.

### Viral plus-strand DNA assay

(i) Infection of C8166 by HIV-1_IIIB_. In a 6-well plate, 5 × 10^6^ C8166 cells were infected by HIV-1_IIIB_ (M.O.I. = 0.5) with serial dilutions of SJP-L-5 (50 μM, 5 μM, and 0.5 μM,) or NVP (1000 nM, 100 nM, and 10 nM) in a final volume of 8 mL RPMI-1640 media containing 10% FBS and 1 μM RAL. RAL was used to prevent viral DNA integration into host chromosomes. The plate was then incubated in a humidified CO_2_ incubator at 37 °C for 48 h. (ii) Hirt DNA preparation. The unintegrated viral DNA was extracted following Hirt’s methods^30,31^. (iii) Southern blot. All DNA samples were digested by *Pst* I (Fermentas, China). Following denaturation in boiling water, the DNA was immediately cooled in ice/water. DNA was then subjected to electrophoresis in 0.7% agarose gel in 0.5× TBE and analyzed by DIG-labelled chemiluminescent Southern blot according to manufacturer’s instructions (DIG-high prime DNA labeling and detection starter kit II, Roche). The probes used here were a double-stranded HIV-1 DNA fragments within the RTase gene(from the position 2096 to 3375 of HIV-1_IIIB_ genome, GenBank accession number: EU541617), which hybridized to both the minus and the upstream plus strands, or a single-strand DNA fragment within the gp41 gene (from the position 6296 to 6886 of HIV-1_IIIB_ genome), which hybridized to the downstream plus strand^5^. The chemiluminescent signal was recorded on X-ray film (Kodak, China). The gray value of the film was analyzed via ImageJ 1.46r software.

### Selection of SJP-L-5-resistant variant viruses

The viruses resistant to SJP-L-5 were selected by adding the compound progressively to HIV-1_IIIB_-infected C8166 cells, according to previously described methods^32^. Briefly, HIV-1_IIIB_-infected C8166 cells were cultured with 0.9 μM SJP-L-5. Every ~7 days, when syncytia formation was observed to be over 80%, a doubled concentration of SJP-L-5 was added until the compound reached a concentration of 122 μM.

### Genotypic resistance assays

SJP-L-5-resistant virus variants were used to analyze the phenotypic and genotypic resistance features of the compound. The RNA of the resistant virus was extracted and reverse transcribed to cDNA. RTase gene of the cDNA was amplified by PCR as previously reported^33^. A full-length RTase-encoding sequence of 1680 bp was cloned, and positive clones were selected. After sequencing analysis, the gene was aligned with the wild-type HIV-1_IIIB_ strain sequence (GenBank accession number: EU541617). Mutation frequency was then calculated.

### Construction of site-directed mutants (SDM) for phenotypic resistance assays

SDM were constructed based on the HIV-1 infectious clone pNL4-3. Mutagenesis was carried out by using the Fast mutagenesis system kit (TransGen, China) according to manufacturer’s instructions. V108I, E138K, and Y181C were introduced into the RTase gene. These mutant plasmids were transfected into C8166 cells using TurboFect (Fermentas, China) according to manufacturer’s instructions. The supernatant of each mutated virus was collected and stored at −70 °C.

### Antiviral activity of SJP-L-5 against resistant viruses and site-directed mutants

Nucleoside resistant HIV-1 strains (HIV-1_A018_ and HIV-1_74V_), non-nucleoside resistant HIV-1 strains (HIV-1_A17_ and HIV-1), and RTase mutant strains (V108I, E138K, and Y181C) were used. The antiviral activity assay was performed as previously described^34^.

### Statistical analysis

The results were expressed as the mean ± standard deviation (SD). Statistical significance was determined with Student’s *t*-test using the SPSS 17.0 software. Differences were significant at *P* < 0.05.

## Electronic supplementary material

Supplementary Materials
