# Supplementary material for: SJP-L-5 inhibits HIV-1 polypurine tract primed plus-strand DNA elongation, indicating viral DNA synthesis initiation at multiple sites under drug pressure
Source: Sci Rep. 2018 Feb 7;8:2574. doi: 10.1038/s41598-018-20954-5 (PMC5803243; doi:10.1038/s41598-018-20954-5)
Supplement: Supplementary file 1 — Supplementary Materials [file 41598_2018_20954_MOESM1_ESM.pdf]

Supplementary Materials

Title: SJP-L-5 inhibits HIV-1 polypurine tract primed plus-strand DNA elongation, indicating viral DNA synthesis initiation at multiple sites under drug pressure

Authors: Xing-Jie Zhang[1,2,3#], Rui-Rui Wang[1,4#], Huan Chen[1,3], Rong-Hua Luo[1], Liu-Meng Yang[1], Jing-Ping Liu[5], Han-Dong Sun[5], Hong-Bin Zhang[2], Wei-Lie Xiao[2,5]*, Yong-Tang Zheng[1,3,6]*

Affiliations: [1] Key Laboratory of Bioactive Peptides of Yunnan Province/Key Laboratory of Animal Models and Human Disease Mechanisms of the Chinese Academy of Sciences, Kunming Institute of Zoology, Chinese Academy of Sciences, Kunming, Yunnan 650223, China.

[2] Key Laboratory of Medicinal Chemistry for Natural Resource, Ministry of Education and Yunnan Province, Yunnan University, Kunming, Yunnan 650091, China.

[3] Kunming College of Life Science, University of Chinese Academy of Sciences, Kunming, Yunnan 650204, China.

[4] College of Pharmaceutical Science, Yunnan University of Traditional Chinese Medicine, Kunming, Yunnan 650500, China.

[5] State Key Laboratory of Phytochemistry and Plant Resources in West China, Kunming Institute of Botany, Chinese Academy of Sciences, Kunming, Yunnan 650201, China.

[6] KIZ-SU Joint Laboratory of Animal Models and Drug Development, College of Pharmaceutical Sciences, Soochow University, Suzhou, Jiangsu 215006, China.

*To whom correspondence should be addressed: xiaoweilie@ynu.edu.cn (W.-L. Xiao), phone: +86-871-65031119; zhengyt@mail.kiz.ac.cn (Y.-T. Zheng), phone/fax: +86-871-65195684.

[#]These authors contributed equally to this work.

a) Double Stranded Probe

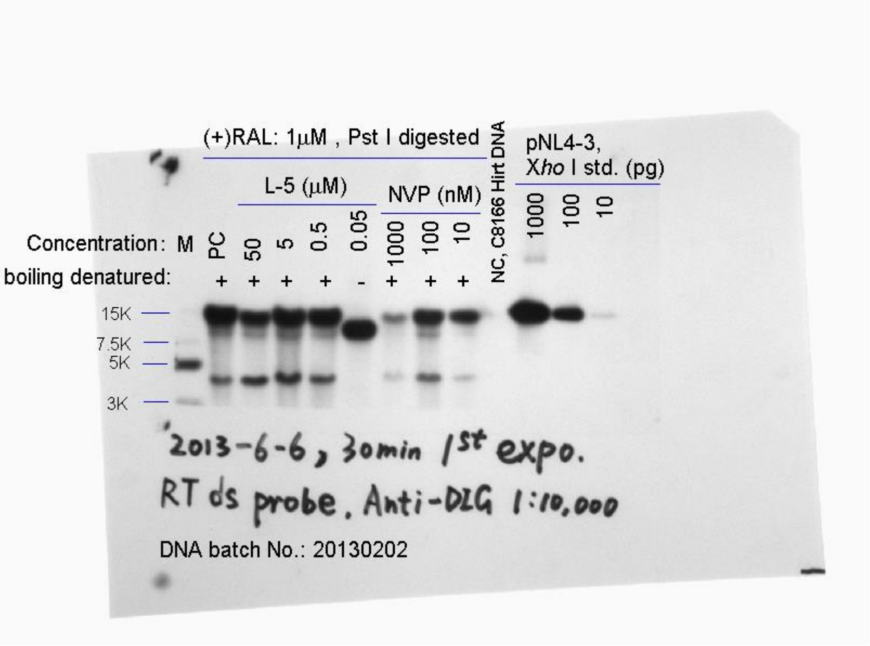

b) Plus-strand Specific Probe

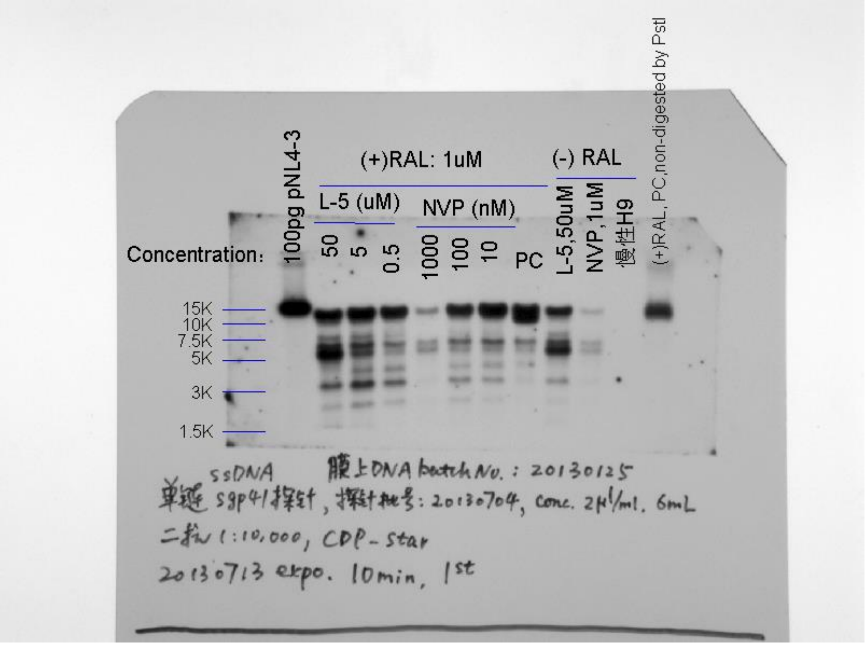

Figure S1. Uncropped Southern blot images. *a*) The full length blot of **Figure 5a.** *b*) The full length blot of **Figure 5b**.
